# Hsa_circ_0001982 promotes the proliferation, invasion, and multidrug resistance of osteosarcoma cells

**DOI:** 10.1002/jcla.24493

**Published:** 2022-05-16

**Authors:** Bochuan Lin, Jing Nan, Kai Lu, Yi Zong, Wencan Fan

**Affiliations:** ^1^ Department of orthopedic surgery Daqing Oilfield General Hospital Daqing Heilongjiang China; ^2^ Department of statistics Long Nan Hospital Daqing Heilongjiang China; ^3^ Department of vascular surgery Daqing Oilfield General Hospital Daqing Heilongjiang China

**Keywords:** hsa_circ_0001982, miR‐143, molecular sponge, multidrug resistance, osteosarcoma

## Abstract

**Background:**

Osteosarcoma (OS) is the most common bone cancer mostly seen in people aged 10–25 years. This research aims to clarify the function of hsa_circ_0001982 in osteosarcoma (OS) and its effect on drug resistance, preliminarily exploring its mechanism.

**Methods:**

The expression of hsa_circ_0001982 and miR‐143 in OS clinical tissues and cells was detected by real‐time fluorescence quantitative polymerase chain reaction (qRT‐PCR), MTT, colony formation assay, and transwell assay assessed cell proliferation, colony formation, migration, and invasion, respectively. The targeted relationship of hsa_circ_0001982 and miR‐143 was verified by a dual‐luciferase reporter assay.

**Result:**

The expression of hsa_circ_0001982 was significantly increased in OS tissues and cells (MG63), as in well as chemoresistant OS tissues and cells (MG63/Dox). Overexpression of hsa_circ_0001982 promoted proliferation, colony formation, migration, invasion, and multidrug resistance in MG63 cells. By contrast, knockdown of hsa_circ_0001982 markedly reduced the resistance of MG63/Dox cells to doxorubicin (IC50 evidently reduced). Bioinformatic prediction showed that miR‐143 was a target miRNA of hsa_circ_0001982, and a dual‐luciferase reporter assay proved this. Further experiments revealed that miR‐143 expression was notably downregulated in OS tissues, chemoresistant OS tissues, and MG63/Dox cells. Moreover, miR‐143 was negatively correlated with hsa_circ_0001982 in OS cells and tissues.

**Conclusion:**

The regulation of malignant behaviors such as proliferation, invasion, migration, and multidrug resistance of OS cells by hsa_circ_0001982 may be achieved by targeting miR‐143. Moreover, hsa_circ_0001982 is a potential target for early diagnosis and targeted therapy of OS.

## Graphical abstract

Has_circ_0001982 was significantly overexpressed in OS tissues and cells, as well as chemotherapy‐resistant OS tissues and cells. Functionally, hsa_circ_0001982 can promote the development of OS cell proliferation, colony formation, invasion, migration, and multidrug resistance, which may be achieved by targeted inhibition of miR‐143 expression. Therefore, hsa_circ_0001982 can be used as a potential target for early diagnosis and targeted therapy of OS.

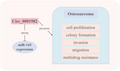

## 1 INTRODUCTION

Osteosarcoma (OS) is the most common bone cancer mostly seen in people aged 10–25 years with some 51 million annual new each year. It usually metastasizes to the lung via the blood transport route at an early stage and results in high mortality.^1^, ^2^, ^3^, ^4^ At present, surgical resection of the lesion, combined with preoperative and postoperative adjuvant therapy, is the first‐line treatment for OS, which can prolong the survival of OS patients. However, the treatment effect for patients with metastatic and recurrent OS is not optimistic leaving a low 5‐year survival rate.^5^ Moreover, the advent of multidrug resistance (MDR) has also become a major challenge to tackling OS, making cancer cells resistant not only to single cytotoxic drugs, but also to a range of drugs with different structures and cellular targets.^6^, ^7^ Therefore, on the one hand, a more definite molecular regulatory mechanism of malignant biological behavior of OS cells and multidrug resistance is required. On the other hand, it is urgent to explore new and effective biomarkers for improving the diagnosis, treatment, and prognosis of OS.

Circular RNAs (circRNAs) were originally found to be novel closed circular non‐coding RNA molecules stably present in mammals, which are composed of both exon splicing and intron splicing, without a 5′ end cap and a 3′ end tail.^8^, ^9^ However, recent studies have shown that in some cancers, circRNA has translation function and can encode proteins and peptides.^10^ CircRNAs contain binding sites for multiple microRNAs (miRNAs) and can enrich miRNAs. They can also regulate the expression levels of target genes by interfering with the binding of miRNAs to target genes, so as to participate in the occurrence and development of a variety of diseases, and thus are considered a potential disease marker.^11^ Numerous studies have demonstrated that circRNAs act as oncogenes or suppressors in the development and progression of a variety of tumors.^12^, ^13^, ^14^ Of note, some circRNA molecules such as circ‐NT5C2 and circ100876 are involved in malignant behaviors such as proliferation and invasion of OS.^15^, ^16^

Hsa_circ_0001982 (circRNA RNF111), as a member of circRNAs, plays a role in a variety of malignancies. For example, Qiu et al. found that hsa_circ_0001982 was upregulated in breast cancer (BC) tissues and cells under hypoxia, and its high expression promoted glycolysis, cell viability, migration, and invasion of BC cells.^17^, ^18^ Besides, its noticeably high expression was also discovered in colorectal cancer^19^ and gastric cancer^20^ tissues and cells where it promoted the malignant behavior of tumors and served as a potential tumor marker. At the same time, hsa_circ_0001982 was also found to be associated with chemoresistance of malignant tumors. For example, hsa_circ_0001982 was distinctly highly expressed in adriamycin‐resistant^21^ and paclitaxel‐resistant BC tissues and cells, and its high expression promoted paclitaxel resistance in BC.^22^ However, there is no study on the role and mechanism of hsa_circ_0001982 in OS malignant progression and its multidrug resistance. Therefore, in this study, function experiments were performed to explore the expression of this circRNA in OS, what effects it has on OS cell biological functions such as proliferation, colony formation, migration, invasion, and multidrug resistance, and how it affects. This provides some rationale for subsequent studies and clinical treatment of OS.

## 2 MATERIALS AND METHODS

### 2.1 Clinical samples

Twenty pairs of tumor tissues and histologically normal tissues (Normal) were collected from OS patients who did not receive chemoradiotherapy in our hospital, comprising 10 tumor tissues (Sensitive) from chemosensitive OS patients and 10 (Resistance) from chemoresistant OS patients. All tissue samples were confirmed by the Department of Pathology of the hospital. The study was approved by Daqing Oilfield General Hospital Ethics Committee (ZYAF/SC‐07/02.0), and all participating patients signed the informed consent form.

### 2.2 Cell culture and transfection

Both osteoblast cell line hFOB1.19 and OS cell line MG63 were purchased from the Shanghai Institute of Cell biology, China. The doxorubicin‐resistant OS cell line MG63/Dox was established by culturing drug‐sensitive MG63 cells in a medium with gradually increased concentrations of doxorubicin, also known as adriamycin. Then, hFOB1.19, MG63, and MG63/Dox cells were cultured with DMEM medium (Gibco, USA) containing 10% fetal bovine serum (FBS, Gibco, USA) and 1% penicillin streptomycin at 37°C, 5% CO_2_, and 95% humidity, and the medium was changed every 2 days.

Hsa_circ_0001982 interference fragment (si‐hsa_circ_0001982) and control (si‐NC), miR‐143 mimics and control (NC mimic), hsa_circ_0001982 overexpression plasmid (hsa_circ_0001982), and control (Vector) were designed and synthesized by GenePharma (Shanghai, China). Cells were cultured to log phase, digested, diluted to 2 × 10^6^ cells/ml, and seeded in 6‐well plates. When confluence reached 70%–90%, the above fragments or vectors were transfected into the cells by Lipofectamine 2000 (Invitrogen, USA), and the cells were collected after 48 h of culture.

### 2.3 Real‐time fluorescence quantitative polymerase chain reaction (qRT‐PCR)

Total RNA was extracted from cells or tissues using Trizol reagent (Invitrogen, USA) and reversely transcribed into cDNA according to the instructions of the reverse transcription‐PCR kit (Takara, Japan). A SYBR Green PCR kit (Takara, Japan) was used to detect the relative expression levels of hsa_circ_0001982, RNF111, and miR‐143, with GAPDH and U6 as internal references, respectively. The relevant primer sequences are listed in Table 1, and the data were processed using the 2 ^− ΔΔCt^ method.^23^

**TABLE 1:** Primer Sequences for qRT‐PCR

Gene	Sequence (5′ to 3′)
Hsa_circ_0001982	F: ACAATCCAGCTGTTCCCTCA
	R: GGTGCATCAGAAGGAATCTCA
miR‐143	F: ACACTCGAGCTGGGGCTTCTCCTGGCTCTCC
	R: TGGTGTGGTGGAGTCG
U6	F: CGCTTCGGCAGCACATATAC
	R: TTCACGAATTTGCGTGTCAT
GAPDH	F: GCCATCACAGCAACACAGAA
	R: GCCATACCAGTAAGCTTGCC

### 2.4 MTT assay

#### 2.4.1 Cell proliferation assay

Cells in the logarithmic phase were collected and adjusted to a concentration of 5 × 10^4^ cells/ml. Next, the cells were mixed well to inoculate 100 μl to a 96‐well plate. After the cells had completely adhered and cultured for 24 h, a proliferation assay was carried out according to the instructions of the MTT kit. Briefly, 10‐μl MTT solution (5 mg/mL) was added to each well for a 4‐h culture. Then, the supernatant was aspirated and 110‐μl formazan solution was added to each well and shaken at low speed for 10 min for full dissolution. Then, the absorbance value at 490 nm was detected by a microplate reader and the cell proliferation rate was calculated. And, six replicate wells were set for each group.

#### 2.4.2 Determination of drug Sensitivity and cytotoxicity

Cells at the logarithmic phase were collected and adjusted to a concentration of 5 × 104 cells/ml and inoculated 100 μl to a 96‐well plate for a 12‐h culture. The cells were mixed with 10 μl of paclitaxel, cisplatin, methotrexate, and doxorubicin/adriamycin at different concentrations for a 48‐h culture. Then, 10 μl of MTT solution (5 mg/ml) was added to each well and cultured for 4 h. The supernatant was removed, and 110 μl of formazan solution was added to each well and shaken at low speed for 10 min for full dissolution. Five duplicate wells were set for each group and microplate reader determined the absorbance at 490 nm. Finally, the half inhibitory concentration (IC50) was calculated.

### 2.5 Cell colony formation assay

Cells in logarithmic growth were collected and the density of the cell suspension was adjusted to 1 × 10^3^ cells/ml. Then, the cells were seeded into a culture dish containing DMEM medium and cultured statically at 37°C in 5% CO_2_ for about 14 days. For a further step, crystal violet solution (Beyotime, China) was added to each well for staining the cell clones, and finally, photographs were taken for recording.

### 2.6 Transwell assay

First 100 μl of diluted Matrigel (BD Biosciences, USA) was added to Transwell insert (Corning, USA) and allowed to solidify for 3–5 h in a 37°C incubator. Then, cells were suspended in a serum‐free medium to achieve a concentration of 5 × 10^5^ cells/mL. Next, 100 μl of resuspended cells was added to the upper chamber with or without Matrigel, and 600 μl of DMEM medium containing 20% FBS to the lower chamber. After culture for 18–24 h, 4% paraformaldehyde (Beyotime, China) was utilized for fixing the cells for 30 min and crystal violet solution for 30 min staining. The excess staining solution was washed off with PBS and cells in the upper chamber of the transwell were wiped off. Subsequently, after the insert was dried, photos were taken for recording under a microscope.

### 2.7 Dual‐luciferase reporter assay

CircInteractome (https://circinteractome.nia.nih.gov/) predicted the binding site between hsa_circ_0001982 and miR‐143. The hsa_circ_0001982 wild‐type (hsa_circ_0001982‐wt) or mutant (hsa_circ_0001982‐mut) sequences containing the binding sequence were constructed into a dual‐luciferase reporter plasmid (GP‐miRGLO, GenePharma). And, 293 T cells were seeded in 6‐well plates and transfected with the luciferase reporter plasmid and miR‐143 mimics or NC mimics until the confluency reached 80%–90%. The luciferase activity was measured by a dual‐luciferase reporter kit (Promega, USA) after 48 h of culture.

### 2.8 Statistical analysis

Experimental data were expressed as mean ± standard deviation (SD), and statistical analysis was performed using SPSS 23.0 software. Comparison between two groups was conducted by using t test, and comparison of multiple groups by one‐way analysis of variance. *p* < 0.05 indicates a significant difference.

## 3 RESULTS

### 3.1 Hsa_circ_0001982 is upregulated in OS tissues and cells

**FIGURE 1:**
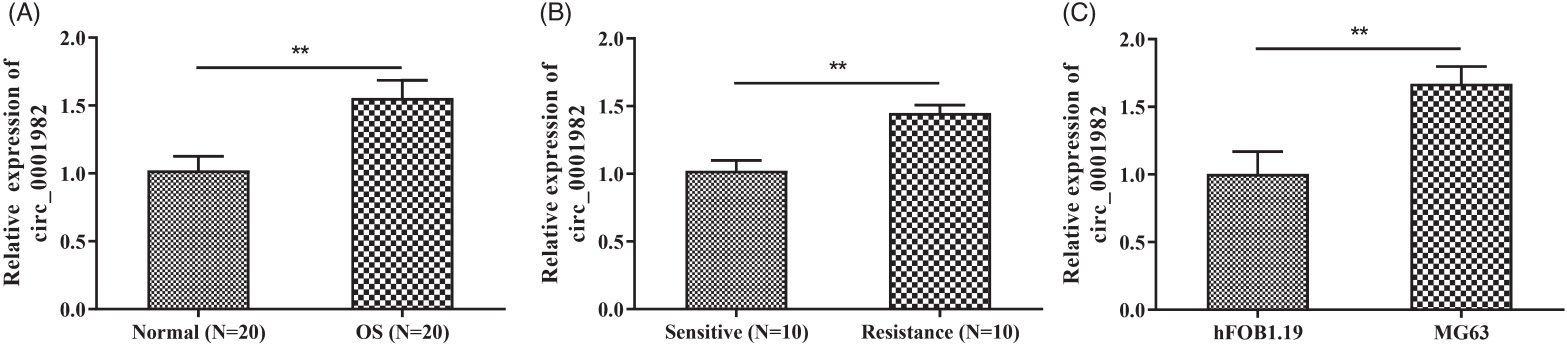
Hsa_circ_0001982 is upregulated in OS tissues and cells. (A‐C) qRT‐PCR for detecting the expression of hsa_circ_0001982 in OS and Normal tissues (A), in chemosensitive and chemoresistant OS tissues (B), and in osteoblast cell line hFOB1.19 and OS cell line MG63 (C); **p < 0.01

To explore the role of hsa_circ_0001982 in the development of OS and chemoresistance, the expression of hsa_circ_0001982 in OS and doxorubicin‐resistant OS tissues and cell lines was detected by qRT‐PCR. Upregulated expression of hsa_circ_0001982 was observed in OS tissue (OS), OS resistant tissue (Resistance), and OS cell line MG63 (*p* < 0.01) (Figure 1A‐C). These results suggested that hsa_circ_0001982 might be associated with the progression of OS and chemoresistance.

### 3.2 Hsa_circ_0001982 promotes MG63 cell proliferation, invasion, and migration

**FIGURE 2:**
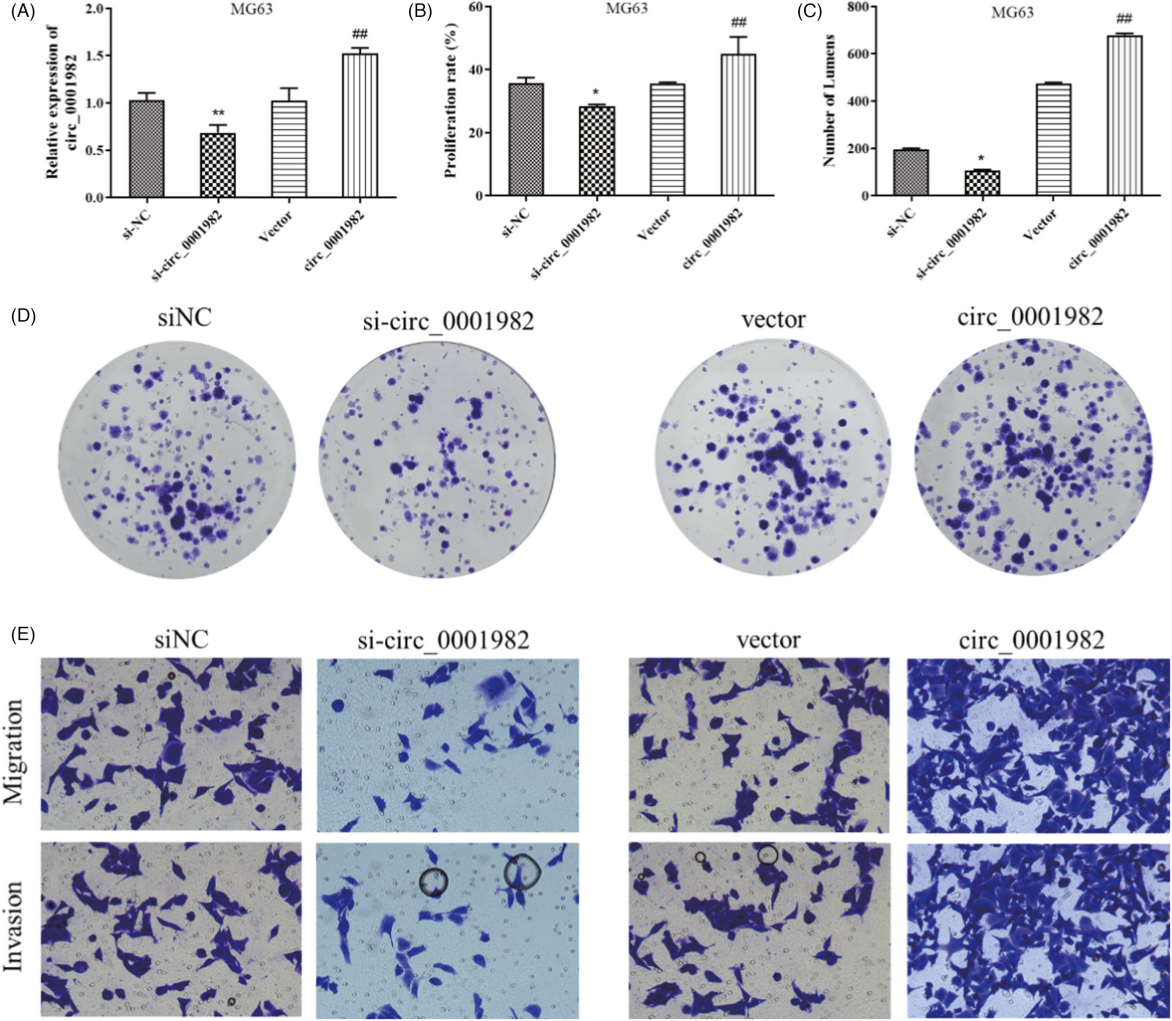
Hsa_circ_0001982 promotes MG63 cell proliferation, invasion, and migration. (A) qRT‐PCR was used to detect the expression level of hsa_circ_0001982 in MG63 cells after transfection; (B) MTT was utilized to determine the proliferation level of MG63 cells; (C‐D) Colony formation assay was performed for detecting colony formation of MG63 cells; E: Transwell was carried out to test migration and invasion of MG63‐transfected cells, *p < 0.05 and **p < 0.01 vs., si‐NC group; ##*p* < 0.01 vs., vector

To further explore the function of hsa_circ_0001982 in OS, hsa_circ_0001982 in MG63 cells was knocked down and overexpressed, respectively. As shown in Figure 2A, qRT‐PCR results confirmed successful transfection. Mainly, knockdown of hsa_circ_0001982 notably reduced the proliferation rate, cell viability, invasion, and migration ability of MG63 cells (*p* < 0.05); however, such malignant behaviors were significantly increased in the presence of overexpressed hsa_circ_0001982 (*p* < 0.01) (Figure 2B‐E). The above results indicated that hsa_circ_0001982 could promote OS cell proliferation, migration, and invasion.

### 3.3 Hsa_circ_0001982 improves multidrug resistance of MG63 Cells

**FIGURE 3:**
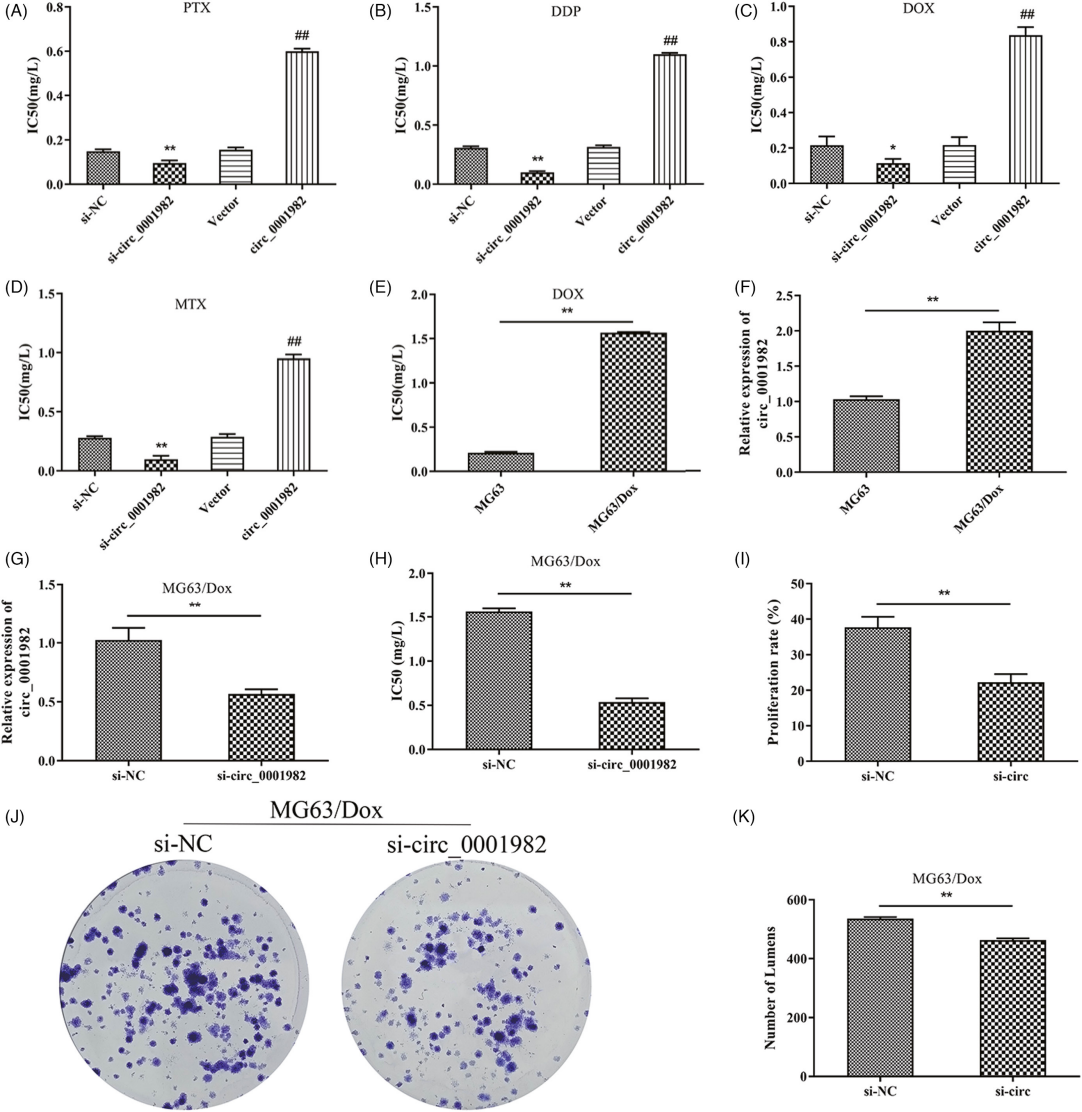
Hsa_circ_0001982 improves multidrug resistance in MG63 cells. (A‐D) IC50 of transfected MG63 cells after treatment with paclitaxel (A), cisplatin (B), doxorubicin/adriamycin (C), and methotrexate (D); E: IC50 of MG63 and MG63/Dox cells under doxorubicin treatment; F‐G: qRT‐PCR for detecting the expression level of hsa_circ_0001982 in MG63 and MG63/Dox cells (F), and in transfected MG63/Dox cells (G); H: IC50 of transfected MG63/Dox cells under doxorubicin treatment; I: MTT assay for checking proliferation rate of transfected MG63/Dox cells under doxorubicin treatment; (J‐K) Cell cloning assay for testing colony formation ability of transfected MG63/Dox cells under doxorubicin treatment; *p < 0.05 vs., si‐NC, **p < 0.01 vs., si‐NC

Paclitaxel, cisplatin, doxorubicin, and methotrexate are chemotherapy drugs commonly used in OS clinic trials.^24^ To investigate the relationship between hsa_circ_0001982 and chemoresistance in OS, we administered these drugs to OS cells in different groups and detected the viability by MTT assay. The results displayed that as chemotherapy greatly decreased the IC50 in the control groups, additional treatment with si‐hsa_circ_0001982 further reduced the concentration (*p* < 0.05), while overexpression of hsa_circ_0001982 could noticeably increase the IC50 of MG63 cells (*p* < 0.01) (Figure 3A‐D). That meant hsa_circ_0001982 could improve the resistance of MG63 cells to a variety of chemotherapeutic drugs.

Further validation of the function of hsa_circ_0001982 in cell resistance showed that the IC50 of doxorubicin‐resistant cell line MG63/Dox was increased by about 6.5‐fold compared with that in MG63 cells (Figure 3E) as the expression of hsa_circ_0001982 was significantly higher in the former (*p* < 0.01, Figure 3F). Further, the level of hsa_circ_0001982 in MG63/Dox cells was successfully knocked down by si‐hsa_circ_0001982 transfection (Figure 3G). Functional experiments revealed that si‐hsa_circ_0001982 obviously reduced the proliferation and viability of MG63/Dox cells and resulted in a significant decrease in the IC50 of MG63/Dox cells under doxorubicin treatment (*p* < 0.01) (Figure 3H‐K). Collectively, hsa_circ_0001982 significantly improved the resistance of OS cells to a variety of chemotherapeutic drugs.

### 3.4 Hsa_circ_0001982 targets miR‐143 expression in OS tissues and cells

**FIGURE 4:**
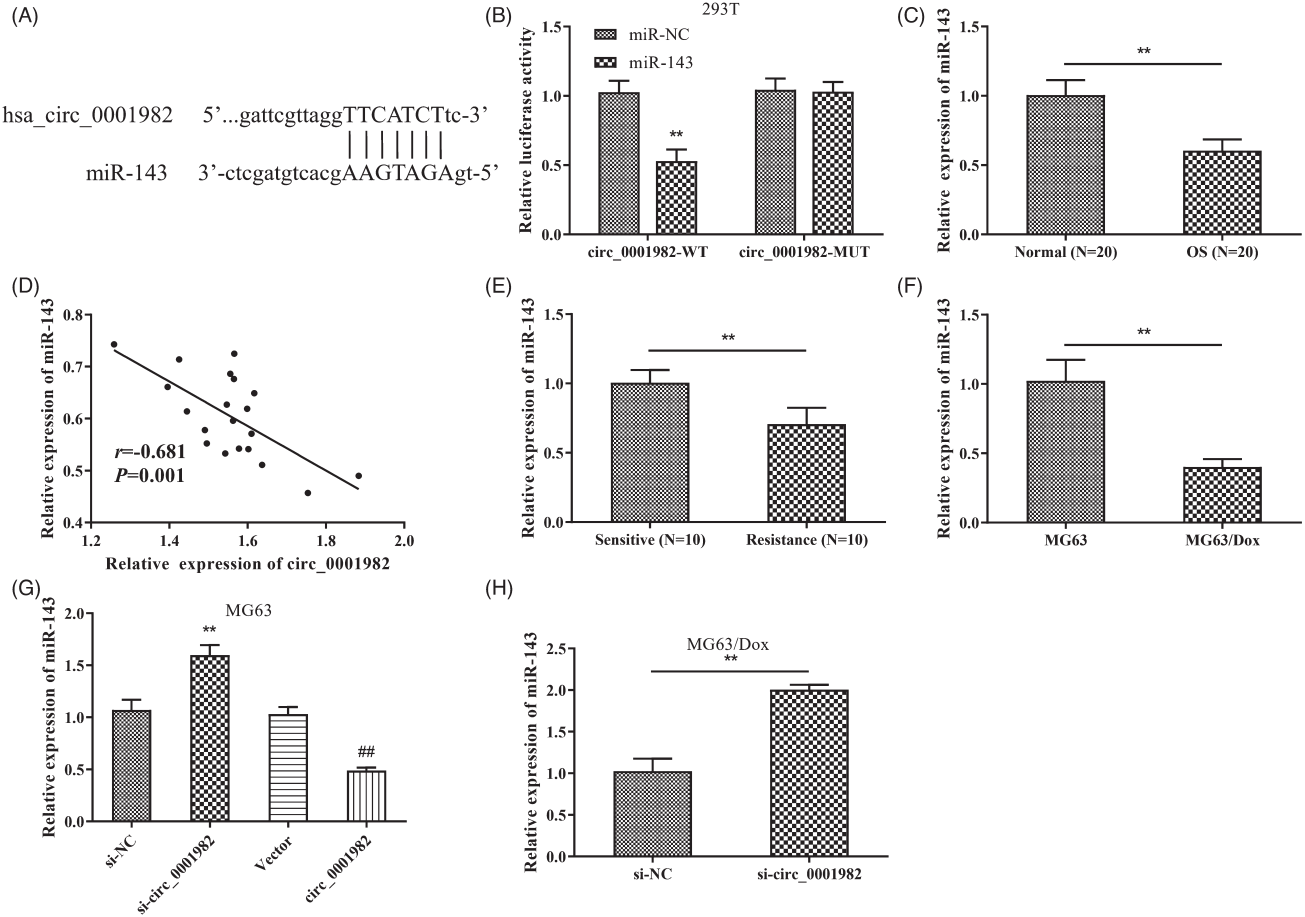
Hsa_circ_0001982 targets and inhibits the expression of miR‐143. (A) Bioinformatics predicted the target sequences of hsa_circ_0001982 and miR‐143 (CircInteractome); (B) Dual‐luciferase reporter assay validated hsa_circ_0001982 targeted miR‐143; (C) qRT‐PCR was performed to detect the expression levels of miR‐143 in OS and normal tissues; (D) Correlation between hsa_circ_0001982 and miR‐143 expression in OS tissues; E‐H: qRT‐PCR was utilized to check the expression levels of miR‐143 in chemosensitive and chemoresistant OS tissues (E), in MG63 and MG63/Dox cells (F), and in transfected MG63 cells (G) and transfected MG63/Dox cells (H). **p < 0.01 vs., si‐NC group, ##*p* < 0.01 vs., vector group

To further explore the regulatory mechanism of hsa_circ_0001982 in OS, we identified miR143 with complementary base matches to hsa_circ_0001982 by performing CircInteractome (Figure 4A). The results of the luciferase reporter assay demonstrated that co‐transfection of miR‐143 mimics greatly inhibited the luciferase activity of the hsa_circ_0001982‐WT vector but had no effect on the hsa_circ_0001982‐MUT vector (Figure 4B), validating hsa_circ_0001982 targeted miR‐143. Besides, relatively low expression of miR‐143 expression was found in OS and chemoresistant OS tissues and cells, and its expression level was negatively correlated with hsa_circ_0001982 expression in OS tissues (Figure 4C‐F). In addition, si‐hsa_circ_0001982 evidently upregulated miR‐143 expression in MG63 and MG63/Dox cells, while miR143 expression was markedly decreased after overexpression of hsa_circ_0001982 in MG63 cells (Figure 4G‐H). The above results indicated that circ‐0001982 could directly bind to miR‐143 and negatively regulate miR‐143 expression.

## 4 DISCUSSION

Despite great progresses in the early diagnosis and treatment of OS, most patients still suffer from metastasis and chemoresistance and the etiology and pathogenesis of OS remain unclear.^25^ circRNAs are highlighted to play an important role in a variety of human diseases, such as cancer, neurological diseases, and atherosclerosis.^26^ Of them, hsa_circ_0001982 is highly expressed in BC, gastric cancer, and colorectal cancer tissues and cells, and promotes the occurrence of malignant behaviors such as proliferation and invasion of tumor cells.^17^, ^18^, ^19^, ^27^ Our study also found that the expression of hsa_circ_0001982 was significantly increased in OS tissues and cells, suggesting its oncogene role in OS. Cell function assays consistently revealed that overexpression of hsa_circ_0001982 promoted malignant phenotypes such as proliferation, colony formation, migration, and invasion. These results further demonstrated that hsa_circ_0001982 serves as a pro‐oncogene in OS.

Besides, we found that hsa_circ_0001982 was upregulated in chemoresistant OS tissues and chemoresistant OS cell line MG63/Dox, indicating that its expression might be associated with chemoresistance in OS. Further experiments revealed that si‐hsa_circ_0001982 weakened the resistance of MG63 to paclitaxel, cisplatin, doxorubicin, and methotrexate, while overexpressed hsa_circ_0001982 induced the multidrug resistance. Moreover, knockdown of hsa_circ_0001982 caused decreases in MG63/Dox cell proliferation and colony formation, leaving cells less resistance of the cells to doxorubicin treatment. These results suggested that overexpression of hsa_circ_0001982 could improve the chemoresistance of OS cells, consistent with findings by Li and Zang et al. that hsa_circ_0001982 improves the resistance of BC cells to multiple chemotherapeutic drugs.^21^, ^22^ Collectively, hsa_circ_0001982 plays an integral role in the development of multidrug resistance in tumors.

Circular RNAs can sponge miRNAs to affect the expression of downstream target genes of miRNAs and further the progression of a variety of cancers.^28^, ^29^ Hsa_circ_0001982, for instance, can promote the occurrence and progression of malignant tumors by targeting miR‐144, miR‐1287‐5p, miR‐27b‐3p, and miR‐140‐5p.^17^, ^19^, ^22^, ^30^ We found that hsa_circ_0001982 was able to target miR‐143 by bioinformatics prediction, which was confirmed by dual‐luciferase reporter assay. Furthermore, qRT‐PCR results displayed a negative correlation between the expression of hsa_circ_0001982 and miR‐143 in OS tissues. MiR‐143 is a class of single‐stranded non‐coding RNAs, which promotes the malignant behavior of OS cells by regulating the expression of target genes such as MAPK7,^31^ PAI‐1,^32^ and Bcl‐2.^33^ Additionally, miR‐143 is also associated with chemoresistance in OS.^34^ In the study by Tang et al., hsa_circ_0001982 functions in BC by regulating miR‐143 expression.^18^ Herein, we speculated that hsa_circ_0001982 may also contribute to the development of malignant biological behavior of OS cells and enhance MG63 multidrug resistance through sponging miR‐143. Nevertheless, we did not experimentally prove whether hsa_circ_0001982 functions as a cancer promoter through miR‐143 in our study. At the same time, the research on the function of hsa_circ_0001982 in OS has only been explored *in vitro* cells, so *in vivo* experiments are needed for further validation.

## 5 CONCLUSIONS

Hsa_circ_0001982 is significantly highly expressed in OS tissues and cells as well as chemoresistant OS tissues and cells. Functionally, hsa_circ_0001982 can promote OS cell proliferation, colony formation, invasion, migration, and the development of multidrug resistance, which may be achieved by targeting inhibition of miR‐143 expression. As a result, hsa_circ_0001982 may be used as a potential target for early diagnosis and targeted therapy of OS.

## CONFLICT OF INTEREST

The authors declared that they have no competing interests.

## Data Availability

The data that support the findings of this study are available from the corresponding author upon reasonable request.
